# Risk factors for aspiration pneumonia after definitive chemoradiotherapy or bio-radiotherapy for locally advanced head and neck cancer: a monocentric case control study

**DOI:** 10.1186/s12885-017-3052-8

**Published:** 2017-01-17

**Authors:** Sadayuki Kawai, Tomoya Yokota, Yusuke Onozawa, Satoshi Hamauchi, Akira Fukutomi, Hirofumi Ogawa, Tsuyoshi Onoe, Tetsuro Onitsuka, Takashi Yurikusa, Akiko Todaka, Takahiro Tsushima, Yukio Yoshida, Yosuke Kito, Keita Mori, Hirofumi Yasui

**Affiliations:** 1 Division of Gastrointestinal Oncology, Shizuoka Cancer Center, 1007 Shimonagakubo, Nagaizumi, Sunto-gun, Shizuoka 411-8777 Japan; 2 Division of Medical Oncology, Shizuoka Cancer Center, Sunto-gun, Shizuoka Japan; 3 Division of Radiation Oncology and Proton Therapy, Shizuoka Cancer Center, Sunto-gun, Shizuoka Japan; 4 Division of Head and Neck Surgery, Shizuoka Cancer Center, Sunto-gun, Shizuoka Japan; 5 Division of Dental and Oral Surgery, Shizuoka Cancer Center, Sunto-gun, Shizuoka Japan; 6 Clinical Research Center, Shizuoka Cancer Center, Sunto-gun, Shizuoka Japan

**Keywords:** Head and neck cancer, Aspiration pneumonia, Risk factor, Chemoradiotherapy, Case–control study

## Abstract

**Background:**

Chemoradiotherapy (CRT) and bio-radiotherapy (BRT) are recognized as standard therapies for head and neck cancer (HNC). Aspiration pneumonia after CRT or BRT is a common late adverse event. Our aim in this study was to evaluate the cause-specific incidence of aspiration pneumonia after CRT or BRT and to identify its clinical risk factors.

**Methods:**

We performed a retrospective analysis of 305 patients with locally advanced HNC treated by CRT or BRT between August 2006 and April 2015.

**Results:**

Of these 305 patients, 65 (21.3%) developed aspiration pneumonia after treatment. The median onset was 161 days after treatment. The two-year cause-specific cumulative incidence by CRT or BRT was 21.0%. Multivariate analysis revealed five independent risk factors for aspiration pneumonia, namely, habitual alcoholic consumption, use of sleeping pills at the end of treatment, poor oral hygiene, hypoalbuminemia before treatment, and the coexistence of other malignancies. A predictive model using these risk factors and treatment efficacy was constructed, dividing patients into low- (0–2 predictive factors), moderate- (3–4 factors), and high-risk groups (5–6 factors), the two-year cumulative incidences of aspiration pneumonia of which were 3.0, 41.6, and 77.3%, respectively. Aspiration pneumonia tended to be associated with increased risk of death, although this was not statistically significant (multivariate-adjusted hazard ratio 1.39, *P* = 0.18).

**Conclusion:**

The cause-specific incidence and clinical risk factors for aspiration pneumonia after definitive CRT or BRT were investigated in patients with locally advanced HNC. Our predictive model may be useful for identifying patients at high risk for aspiration pneumonia.

## Background

Chemoradiotherapy (CRT) is a standard treatment for locally advanced head and neck cancer (HNC) [1]. Radiotherapy (RT) with cetuximab, defined as bio-radiotherapy (BRT), is also considered as a treatment option for patients with locally advanced HNC [2]. Compared with radical surgery, CRT and BRT have an advantage of preserving organ function and patients’ quality of life; however, their toxicities are not less harmful than the risks associated with surgery. In the previous clinical trial RTOG 91–11 [3], non-cancer-related death was more common among patients treated with CRT than with RT alone in a further follow-up, despite the higher rates of laryngeal preservation [4]. This suggests that patients cured by CRT need appropriate management against late toxicity.

Aspiration pneumonia is recognized as pneumonia secondary to the inhalation of food particles, saliva, or gastric acid. Patients with HNC who have undergone definitive CRT tend to have swallowing dysfunction due to mucositis during the treatment period or due to radiation-induced fibrosis of the oropharyngeal musculature after completion of the treatment [5]. Szczesniak et al. [6]. reported that approximately 52% of patients who received RT and 69% who received CRT suffered from dysphasia after treatment, and aspiration pneumonia accounted for 19% of non-cancer-related deaths. Additionally, Xu et al. [7]. suggested that aspiration pneumonia was a poor prognostic factor for patients with HNC who received CRT. Therefore, clinicians should assess the risk of aspiration pneumonia in order to identify patients for whom efforts to prevent it should be implemented.

The purpose of this study was to identify clinical risk factors for aspiration pneumonia after definitive CRT or BRT for patients with advanced HNC. In particular, we focused on the cause-specific incidence of aspiration pneumonia, taking competing events of death and resection of the primary lesion into account.

## Methods

### Study population

Three hundred and forty patients with HNC who received definitive concurrent CRT or BRT at Shizuoka Cancer Center between August 2006 and April 2015 were identified from medical records. Of these, 35 patients with a recurrent or metastatic lesion or resection of the primary lesion before CRT were excluded. Patients with other malignancies were included only if HNC was considered to be the factor most strongly determining their prognosis. Finally, 305 patients were included in this analysis. This study was approved by the Institutional Review Committee of Shizuoka Cancer Center (Shizuoka, Japan) and met the standards set forth in the Declaration of Helsinki.

### Study covariates

We retrospectively collected data on the occurrence of aspiration pneumonia, time to onset of aspiration pneumonia, and overall survival (OS) from the end of treatment. Background covariate candidates for factors predictive of aspiration pneumonia included the following: tumor site, age, gender, Eastern Cooperative Oncology Group (ECOG) performance status, body mass index, TNM staging according to the AJCC/UICC TNM classification, tumor histology, smoking status, habitual alcoholic consumption, distance between the patients’ home and the hospital, family members in the same household, use of proton pump inhibitors (PPIs) or H_2_ blockers, use of angiotensin-converting enzyme (ACE) inhibitors or angiotensin II receptor blockers (ARBs), use of sleeping pills and main feeding at the end of the treatment, presence of gastrostomy during the treatment, oral hygiene, serum albumin (ALB) and hemoglobin (Hb) levels before treatment, coexistence of other malignancies before treatment, and Charlson comorbidity index. We defined habitual alcoholic consumption as the drinking of alcohol four or more days a week, and poor oral hygiene as the presence of moderate or more severe dental plaque assessed by a dentist and/or a dental hygienist. Charlson comorbidity index is a tool for predicting mortality by classifying or weighting comorbidities [8].

We also collected the following treatment-related covariate data: presence or absence of induction chemotherapy, chemotherapy regimen, irradiation technique [conventional three-dimensional conformal radiation therapy (3D-CRT) or intensity-modulated radiation therapy (IMRT)], irradiation field, treatment efficacy evaluated according to Response Evaluation Criteria in Solid Tumors ver. 1.1 [complete response (CR) or non-CR], mucositis and dysphagia during treatment evaluated by Common Terminology Criteria for Adverse Events ver. 4.0, and decreases of ALB, Hb, and body weight after treatment.

### Aspiration pneumonia

Because it is sometimes difficult to clearly distinguish aspiration pneumonia from other types of pneumonia, different definitions of aspiration pneumonia were used in previous studies [9–11]. Therefore, in this study, we defined aspiration pneumonia as a clinical condition that met all of the following criteria: (i) Patients had both subjective and objective symptoms suggesting pneumonia. Subjective symptoms included wet cough, sputum, and fever. Objective symptoms included the presence of coarse crackles in the chest, elevated inflammatory markers (e.g. white blood cell count or C-reactive protein), or image findings (e.g. infiltration on a chest X-ray or consolidation in chest computed tomography). (ii) The presence of aspiration was suspected clinically (choking or delayed swallowing) or by endoscopic or video-fluorographic examinations. (iii) No evidence of micro-organisms that cause atypical pneumonia, such as *Legionella* and *Mycoplasma*.

### Statistical analysis

The cause-specific cumulative incidence of aspiration pneumonia was estimated with nonparametric cumulative incidence functions, taking competing events of death and resection of the primary lesion into account. To investigate potential risk factors for aspiration pneumonia, univariate analysis was carried out for all covariates using Fisher’s exact test, and covariates showing statistical significance were further analyzed using a multivariate logistic regression model. To construct a predictive model, we automatically selected covariates extracted from univariate analysis, and compared the goodness-of-fit among many models on the basis of the stepwise Akaike information criterion (AIC) method [12]. The minimum value from the AIC procedure allows us to select appropriate predictive factors to construct an optimal predictive model objectively. The concordance index to evaluate the discriminatory ability of the model was calculated using the final regression model [13].

The OS time was calculated from the date of treatment end to the date of death due to any cause or to the last date of confirmed survival. Survival rates were estimated using the Kaplan–Meier method. To estimate the association of covariates with overall survival, univariate analysis was carried out using the log-rank test. All statistically significant covariates in univariate analysis were analyzed in multivariate analysis using the Cox regression model.

All statistical tests were two-sided, and *P* ≤ 0.05 was considered significant. Statistical analyses were performed using EZR software (Saitama Medical Center, Jichi Medical University, Saitama, Japan) [14].

## Results

Among the 305 patients, 65 (21.3%) developed aspiration pneumonia after CRT or BRT. Patients’ baseline and treatment-related characteristics are summarized in Table 1. The median age of the patients was 65 years (range 19–83) and 95.1% of them had ECOG PS of 0 to 1. Cisplatin, carboplatin, and cetuximab were concurrently used in 77.1, 13.7, and 9.2% of patients, respectively. Seventy-six (24.9%) of the patients received induction chemotherapy, and 87.5% of them were treated with the combination of docetaxel, cisplatin, and fluorouracil. Additionally, 96.0% of all patients had received systematic oral care [15] since initiation of the treatment. Thirty-six (11.8%) patients had coexisting malignancies included multiple primary HNC, esophageal cancer, gastric cancer, prostate cancer, lung cancer, and renal cancer. All of these cancers were found at an early stage by routine endoscopic or computed tomography screening. After definitive CRT or BRT, 30 (9.8%) patients underwent resection of the primary lesion and 45 (14.7%) underwent neck dissection for a residual lesion or recurrence.

**Table 1:** Patients’ characteristics

Background	n (%)
Age	
< 65 years	149 (49)
≥ 65 years	156 (51)
Gender	
Male	266 (87)
Female	39 (13)
ECOG performance status	
0	181 (59)
1	109 (36)
2	12 (4)
3	3 (1)
Body mass index	
< 18.5	46 (15)
18.5–25	199 (65)
≥ 25	60 (20)
Primary site	
Larynx	45 (15)
Nasopharynx	38 (12)
Hypopharynx	112 (37)
Nasal sinus	17 (6)
Oropharynx	79 (26)
Oral cavity	14 (5)
T-classification	
1	28 (9)
2	110 (36)
3	66 (66)
4	101 (33)
N-classification	
0	63 (21)
1	39 (13)
2a	3 (1)
2b	126 (41)
2c	60 (20)
3	14 (5)
Tumor histology	
SCC	287 (94)
Others	18 (6)
Smoking status	
Never	36 (12)
Past	200 (66)
Current	69 (23)
Habitual alcoholic consumption	
Yes	121 (40)
No	184 (60)
Distance from the hospital	
< 10 km	92 (30)
≥ 10 km	213 (70)
Family members in the same household	
Yes	258 (85)
No	47 (15)
Use of ACEi or ARB	
ARB	53 (17)
ACEi	2 (1)
No	250 (82)
Use of PPI or H_2_ blocker	
Yes	163 (53)
No	142 (47)
Oral hygiene before treatment	
Good	100 (33)
Poor	193 (63)
Unknown	12 (4)
Coexistence of other malignancies	
Yes	36 (12)
No	269 (88)
Comorbidity index	
0	233 (76)
≥ 1	72 (24)
Serum albumin before treatment	
Within normal limits	259 (85)
Less than normal range	46 (15)
Hemoglobin before treatment	
Within normal limits	212 (70)
Less than normal range	93 (30)
Use of sleeping pills at the end of treatment	
Yes	210 (69)
No	95 (31)
Main feeding at the end of treatment	
Oral	127 (42)
Non-oral	178 (58)
Presence of gastrostomy during the treatment	
Yes	173 (57)
No	132 (43)
Induction chemotherapy	
Yes	76 (25)
No	229 (75)
Concurrent chemotherapy regimen	
CDDP-based	235 (77)
CBDCA-based	42 (14)
Cetuximab	28 (9)
Radiation technique	
Conventional 3D-CRT	241 (79)
IMRT	64 (21)
Irradiation field	
Primary lesion alone	40 (13)
Hemi neck	19 (6)
Whole neck	246 (81)
Treatment efficacy	
CR	199 (65)
Non-CR	106 (35)
Body weight loss after treatment	
< 10%	178 (58)
≥ 10%	127 (42)
Serum albumin decreasing post-treatment	
< 20%	114 (37)
≥ 20%	191 (63)
Hemoglobin decreasing post-treatment	
< 30%	138 (45)
≥ 30%	167 (55)
The worst mucositis grade during treatment	
0	3 (1)
1	16 (5)
2	102 (33)
3	182 (60)
4	2 (1)
The worst dysphagia grade during treatment	
0	12 (4)
1	84 (28)
2	76 (25)
3	133 (44)
4	0
Resection of primary lesion post-CRT or -BRT	
Yes	30 (10)
No	275 (90)
Neck dissection post-CRT or -BRT	
Radical neck dissection	0
Modified radical neck dissection	1 (1)
Selective neck dissection	44 (14)

*Abbreviations*: *ECOG* Eastern Cooperative Oncology Group, *SCC* Squamous cell carcinoma, *ACEi* Angiotensin-converting enzyme inhibitor, *ARB* Angiotensin II receptor blocker, *PPI* Proton pump inhibitor, *3D-CRT* Three-dimensional conformal radiation therapy, *IMRT* Intensity-modulated radiation therapy, *CR* Complete response, *CRT* Chemoradiotherapy, *BRT* Bio-radiotherapy

The normal range of laboratory data at our institution: Serum albumin (3.8–5.2 g/dl), hemoglobin (male: 13.5–17.6 g/dl, female: 11.3–15.2 g/dl)

**Fig. 1:**
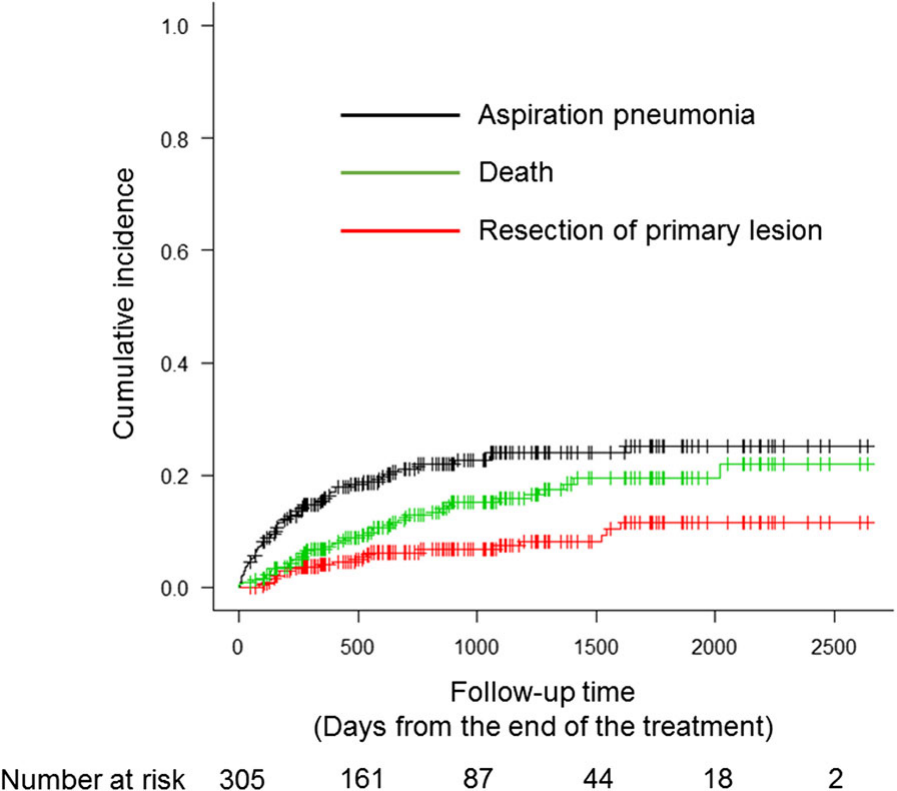
Cumulative incidence of aspiration pneumonia and other competing risks including death and resection of primary lesion. Vertical dashes indicate censored observations

The median time from the end of treatment to aspiration pneumonia events was 161 days (range 3–1623). The median follow-up time was 892 days. The two-year cumulative incidences of aspiration pneumonia and competing events of death and resection of the primary lesion were 21.0% [95% confidence interval (CI) 16.4–26.0%], 12.9% (9.2–17.4%), and 6.2% (3.7–9.5%), respectively (Fig. 1).

Univariate and multivariate analyses identified five independent risk factors for aspiration pneumonia, namely, habitual alcoholic consumption, poor oral hygiene, coexistence of other malignancies, hypoalbuminemia before treatment, and the use of sleeping pills at the end of treatment (Table 2). A difference in the types of sleeping pills (benzodiazepines or others) used was not associated with the onset of aspiration pneumonia (odds ratio 0.95, 95% CI, 0.37–2.39, *P* = 1.00). Of 193 patients with poor oral hygiene before treatment, 135 had been followed up by dentists three months after the treatment. In total, 87 of 135 patients in whom oral hygiene had improved three months after the treatment had a significantly lower frequency of aspiration pneumonia than 48 patients who had poor oral hygiene (18.3% vs. 54.1%, *P* = 0.00003).

**Table 2:** Univariate and multivariate logistic regression analyses for risk factors of aspiration pneumonia

	Univariate analysis		Multivariate analysis	
	Odds ratio (95% CI)	*P*-value	Odds ratio (95% CI)	*P*-value
Age				
< 65 years old	Ref.			
≥ 65 years old	1.71 (0.97–2.99)	0.06		
Gender				
Male	1.06 (0.46–2.43)	0.89		
Female	Ref.			
ECOG performance status				
0–1	Ref.			
2–3	2.61 (0.89–7.62)	0.07		
Body mass index				
< 18.5	Ref.			
18.5–25	0.87 (0.41–1.87)	0.73		
≥ 25	0.71 (0.27–1.83)	0.48		
Primary site				
Larynx	Ref.			
Nasopharynx	1.21 (0.32–4.55)	0.77		
Hypopharynx	2.07 (0.73–5.83)	0.17		
Nasal sinus	1.71 (0.36–8.12)	0.49		
Oropharynx	3.49 (1.23–9.94)	0.02	1.69 (0.50–5.67)	0.39
Oral cavity	4.44 (1.06–18.7)	0.04	1.95 (0.38-9.98)	0.42
T-classification				
1–2	Ref.			
3–4	2.38 (1.32–4.30)	0.004	1.75 (0.85–3.59)	0.12
N-classification				
0–2b	Ref.			
2c–3	1.39 (0.75–2.57)	0.29		
Tumor histology				
SCC	Ref.			
Others	0.20 (0.02–1.57)	0.12		
Smoking status				
Never	Ref.			
Past	0.87 (0.37–2.07)	0.76		
Current	1.14 (0.43–2.98)	0.78		
Habitual alcoholic consumption				
Yes	1.79 (1.00–3.24)	0.05	2.11 (1.01–4.38)	0.04
No	Ref.			
Distance from the hospital				
< 10 km	Ref.			
≥ 10 km	0.88 (0.48–1.59)	0.67		
Family members in the same household				
Yes	Ref.			
No	1.33 (0.64–2.73)	0.44		
Use of ACEi or ARB				
Yes	1.50 (0.76–2.93)	0.23		
No	Ref.			
Use of PPI or H_2_ blocker				
Yes	1.02 (0.58–1.77)	0.94		
No	Ref.			
Oral hygiene before treatment				
Good	Ref.			
Poor	2.63 (1.33–5.21)	0.005	2.81 (1.28–6.16)	0.01
Coexistence of other malignancies				
Yes	2.72 (1.30–5.68)	0.007	3.51 (1.46–8.42)	0.005
No	Ref.			
Comorbidity index				
0	Ref.			
≥ 1	0.59 (0.29–1.22)	0.15		
Serum albumin before treatment				
Within normal limits	Ref.			
Less than normal range	4.60 (2.37–8.95)	0.000006	2.70 (1.12–6.53)	0.02
Hemoglobin before treatment				
Within normal limits	Ref.			
Less than normal range	2.62 (1.49–4.61)	0.0008	1.08 (0.51–2.28)	0.84
Use of sleeping pills at the end of treatment				
Yes	3.22 (1.83–5.67)	0.00005	4.39 (2.21–8.74)	0.00002
No	Ref.			
Main feeding at the end of treatment				
Oral	Ref.			
Non-oral	1.66 (0.93-2.96)	0.09		
Presence of gastrostomy during the treatment				
Yes	2.6 (1.41-4.77)	0.0018	1.58 (0.754-3.31)	0.22
No	Ref.			
Induction chemotherapy				
Yes	1.20 (0.64–2.23)	0.56		
No	Ref.			
Concurrent chemotherapy regimen				
CDDP-based	Ref.			
CBDCA-based	1.01 (0.45–2.25)	0.98		
Cetuximab	1.01 (0.38–2.62)	0.98		
Radiation technique				
Conventional 3D-CRT	1.60 (0.76–3.34)	0.21		
IMRT	Ref.			
Irradiation field				
Primary alone	Ref.			
Hemi neck	3.82 (0.43–33.5)	0.22		
Whole neck	5.43 (0.71–41.6)	0.10		
Treatment efficacy				
CR	Ref.			
non-CR	2.56 (1.46–4.48)	0.0009	1.60 (0.81–3.14)	0.17
Body weight loss after treatment				
< 10%	Ref.			
≥ 10%	1.26 (0.72–2.19)	0.41		
Serum albumin decreasing post-treatment				
< 20%	Ref.			
≥ 20%	1.12 (0.63–1.97)	0.71		
Hemoglobin decreasing post-treatment				
< 30%	Ref.			
≥ 30%	1.31 (0.75–2.29)	0.33		
The worst mucositis grade during treatment				
0–2	Ref.			
3–4	0.91 (0.51–1.58)	0.72		
The worst dysphagia grade during treatment				
0–2	Ref.			
3–4	0.59 (0.33–1.05)	0.07		

*Abbreviations*: *ECOG* Eastern Cooperative Oncology Group, *SCC* Squamous cell carcinoma, *ACEi* Angiotensin-converting enzyme inhibitor, *ARB* Angiotensin II receptor blocker, *PPI* Proton pump inhibitor, *3D-CRT* Three-dimensional conformal radiation therapy, *IMRT* Intensity-modulated radiation therapy, *CR* Complete response

The normal range of laboratory data at our institution: Serum albumin (3.8–5.2 g/dl), hemoglobin (male: 13.5–17.6 g/dl, female: 11.3–15.2 g/dl)

**Fig. 2:**
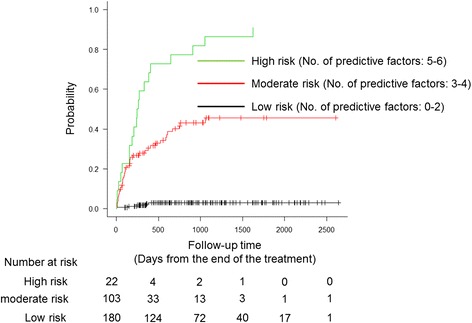
The estimated cumulative incidence of aspiration pneumonia according to the number of predictive factors. Vertical dashes indicate censored observations

Next, we attempted to construct a predictive risk model of aspiration pneumonia from the results of univariate analysis. As a result of AIC stepwise selection, six predictive factors, consisting of the five risk factors extracted from the multivariate analysis and treatment efficacy (non-CR), were selected. Although treatment efficacy was not identified as a statistically significant risk factor, AIC stepwise selection revealed that it was a good predictive factor for the model. This predictive model well divided patients into low- (0–2 factors, *n* = 180), moderate- (3–4 factors, *n* = 103), and high-risk groups (5–6 factors, *n* = 22) by the number of predictive factors, for which the estimated two-year cumulative incidences of aspiration pneumonia were 3.0% (95% CI, 1.1–6.5%), 41.6% (31.0–51.8%), and 77.3% (51.4–90.5%), respectively (Fig. 2). The concordance index was 0.797.

**Fig. 3:**
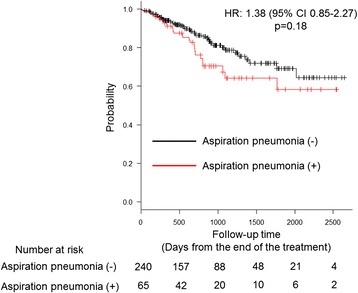
Adjusted Kaplan–Meier curve illustrating overall survival from the date of the end of the treatment among patients with head and neck cancer who received chemoradiation or bio-radiation therapy stratified according to whether or not they developed aspiration pneumonia. Vertical dashes indicate censored observations. CI: confidence interval, HR: hazard ratio

Finally, we investigated the correlation between OS and the occurrence of aspiration pneumonia. Survival curves adjusted for the covariates from a Cox proportional hazard model indicated that the occurrence of aspiration pneumonia tended to be associated with the risk of death, but this was not statistically significant (hazard ratio, 1.39; 95% CI, 0.85–2.27; *P* = 0.18) (Fig. 3).

## Discussion

The important goals of treatment in patients with HNC are not only a cure but also the preservation of quality of life post-treatment. Although approximately 60–70% of patients with HNC treated with CRT suffer from dysphagia or aspiration as a late toxicity [16, 17], in previous studies, the incidence of aspiration pneumonia within a year after CRT was found to differ, ranging from 5.4 to 23% [9, 17, 18]. Furthermore, no differences in the frequency of aspiration pneumonia were seen between the different observation periods, despite the improvement of radiation techniques and general management of CRT over the time. This suggests that various factors other than aspiration are associated with the occurrence of aspiration pneumonia.

To clarify the population at high risk of aspiration pneumonia after CRT or BRT, we investigated the factors predictive of aspiration pneumonia. Several risk factors for aspiration pneumonia in patients with HNC after CRT were reported in previous studies [7, 9, 17]. However, evaluation of the long-term risk factors was often difficult in patients with HNC because these patients’ characteristics varied according to the multimodal therapies that they had received, including surgery, CRT, and RT. In particular, previous studies did not take salvage surgery after CRT into account. Therefore, in these studies, there might not have been accurate estimates of the treatment-specific incidence of aspiration pneumonia after CRT. To our knowledge, the current study is the first regarding specific risk factors and predictive models for aspiration pneumonia as a late toxicity in patients with HNC undergoing definitive CRT or BRT.

We intended to determine risk factors for aspiration pneumonia after CRT or BRT by estimating the cause-specific cumulative incidence. To do this, we first performed cumulative incidence analysis, and regarded resection of the primary lesion as a competing event. Surgical procedures clearly affect swallowing function. For example, total laryngectomy reduces the risk of aspiration and head and neck reconstruction changes patients’ ability to swallow [19, 20]. Therefore, surgical intervention after CRT/BRT may obscure the association of aspiration with CRT or BRT. On the other hand, the effect of neck dissection on aspiration pneumonia has been controversial. For instance, Lango et al. [21]. reported that radical neck dissection (RND) increased the risk of feeding tube dependence in patients with HNC who underwent RT or CRT. On the other hand, Chapuy et al. [22]. reported that types of neck dissection including RND, modified RND, and selective neck dissection (SND) did not aggravate swallowing function. In this study, 45 patients underwent neck dissection, 44 (97%) of which underwent SND. Our analysis suggested no significant association between neck dissection and the occurrence of aspiration pneumonia (*P* = 0.23). Therefore, we did not consider neck dissection as a competing event in cumulative incidence analysis.

Consistent with previous reports [23], hypoalbuminemia was again identified as a factor predictive of aspiration pneumonia after CRT and BRT in our study. The novel predictive factors identified here were poor oral hygiene, use of sleeping pills, coexistence of other malignancies, and habitual alcohol consumption.

Several studies have demonstrated that careful oral management could reduce the risk of aspiration pneumonia in elderly people and patients with a history of cerebral infarction [24, 25]. However, few studies have focused on the correlation between oral hygiene and the risk of aspiration pneumonia in patients with HNC. At our institution, patients with HNC undergoing RT have been routinely referred to dentists and received systematic oral care during the treatment [15]. Indeed, 96.0% of patients received oral evaluation before treatment in this cohort. However, 35.6% of patients initially evaluated as having poor oral hygiene were still assessed as having this same status after the treatment. This suggested that continuous oral management is required in high-risk patients, even after treatment.

Previous studies suggested that sleeping pills increased the risk of aspiration pneumonia [26, 27]. Among these, benzodiazepines were especially associated with the induction of aspiration through gamma-amino-butyric acid type A (GABA-A) signaling in the lesser esophageal sphincter, in addition to inhibition of the central nervous system [28]. However, in our study, benzodiazepines did not specifically increase the risk of aspiration pneumonia more than other sleeping pills. Notably, 83 out of 94 (88.3%) patients who used sleeping pills at the end of the treatment continued to use them even after the treatment. Al-Mamgani et al. [29]. demonstrated that 30.7% of patients with nasopharyngeal cancer who received RT or CRT had the complaint of insomnia during the treatment; however, approximately half of them recovered after the treatment. These findings suggest that the unnecessary administration of sleeping pills might increase the risk of aspiration pneumonia for our patients.

Our data demonstrated that the coexistence of other malignancies was a risk factor for aspiration pneumonia. Of 11 patients who had multiple primary HNC or cervical esophageal cancers simultaneously treated by CRT with main HNC, 7 (63.6%) developed aspiration pneumonia. A previous report suggested that enlargement of the irradiation field increased the risk of aspiration pneumonia [30]. Furthermore, 18 patients underwent surgical or endoscopic resection for esophageal and gastric cancer. Of these, six (33.3%) developed aspiration pneumonia, three of whom developed it within one week post-resection. Therefore, we speculated that post-surgical immunosuppression and anesthesia or sedation before endoscopy might deteriorate swallowing function.

Previous reports indicated that alcohol suppressed the cough reflex, reduced consciousness, and promoted gastro-esophageal reflux [31–33]. Therefore, such complex factors induced by habitual alcohol consumption may be involved in the occurrence of aspiration pneumonia.

Scheld et al. [34]. and Xu et al. [7]. reported that aspiration pneumonia was a significant prognostic factor. Furthermore, Szczesniak et al. [6]. reported that aspiration pneumonia accounted for 19% of non-cancer-related deaths of patients with HNC who received CRT. Therefore, we expected that aspiration pneumonia would be strongly associated with patient survival. However, our study did not show a statistically significant difference in survival between patients who developed aspiration pneumonia and those who did not, probably because of the relatively small number of deaths within the short follow-up period.

Our study had several limitations. First, it involved a retrospective analysis at a single institution. Second, differential diagnosis between aspiration pneumonia and other types of pneumonia was often difficult because the definitions of aspiration pneumonia varied among previous reports [9–11]. Third, the median follow-up of 2.4 years was shorter than in previous studies [4, 7]. The ability of our predictive model might change upon a long-term follow-up. For example, because submucosal remodeling and neurological disturbance slowly progress after irradiation [35], irradiation might have a stronger impact on the occurrence of aspiration pneumonia at a later phase.

Further studies are warranted to validate our predictive model because of the retrospective nature of this study. However, the strength of our study is that almost all patients received standard chemotherapeutic regimens containing platinum or cetuximab, with systematic supportive care such as oral care. Therefore, our predictive model may be more useful for identifying patients at high risk for aspiration pneumonia in recent clinical practice than previous evidences. For example, we propose that clinicians consider swallowing exercises for high- or moderate-risk groups to improve their swallowing function and subsequently prevent aspiration pneumonia [35].

## Conclusions

We investigated the cause-specific incidence and identified risk factors for aspiration pneumonia following definitive CRT or BRT for patients with locally advanced HNC. The prediction of aspiration pneumonia may be necessary to preserve the quality of life and extend life expectancy for patients. Long-term follow-up and further prospective studies are needed to validate the usefulness of our predictive model.

## Abbreviations

3D-CRT: Three-dimensional conformal radiation therapy
ACE: Angiotensin-converting enzyme
AIC: Akaike information criterion
ALB: Serum albumin
ARBs: Angiotensin II receptor blockers
BRT: Bio-radiotherapy
CR: Complete response
CRT: Chemoradiotherapy
ECOG: Eastern Cooperative Oncology Group
GABA-A: Gamma-amino-butyric acid type A
Hb: Hemoglobin
HNC: Head and neck cancer
IMRT: Intensity-modulated radiation therapy
OS: Overall survival
PPIs: Proton pump inhibitors
RND: Radical neck dissection
RT: Radiotherapy
SND: Selective neck dissection
